# Dynamic expression of the myocardial sigma-1 receptor after doxorubicin cardiomyopathy using radioiodine-labeled 2-[4-(2-iodophenyl)piperidino]cyclopentanol (OI5 V) imaging

**DOI:** 10.1007/s12149-025-02062-3

**Published:** 2025-05-29

**Authors:** Zhuoqing Chen, Hiroshi Wakabayashi, Hiroshi Mori, Tomo Hiromasa, Xue Zhang, Takashi Kozaka, Kazuma Ogawa, Seigo Kinuya, Junichi Taki

**Affiliations:** 1 Department of Nuclear Medicine, Kanazawa University Hospital, 13-1 Takara-Machi, Kanazawa, Ishikawa 920-8641 Japan; 2 Division of Probe Chemistry for Disease Analysis, Research Center for Experimental Modeling of Human Disease, Kanazawa University, 13-1 Takara-Machi, Kanazawa, Ishikawa Japan; 3 Graduate School of Medical Sciences, Kanazawa University, Kakuma-Machi, Kanazawa, Ishikawa Japan; 4 Kanazawa Advanced Medical Center, 13-13 Takara-Machi, Kanazawa, Ishikawa Japan

**Keywords:** OI5 V, Sigma-1 receptor, Doxorubicin-induced cardiotoxicity, TGF-β1, Molecular imaging

## Abstract

**Objective:**

We aimed to evaluate the expression of the intensity and distribution of the sigma-1 receptor (σ1R) demonstrated by radiolabeled 2-[4-(2-iodophenyl)piperidino]cyclopentanol (OI5V) in a rat model of doxorubicin-induced cardiotoxicity.

**Methods:**

Wistar rats received doxorubicin (DOX; 2 mg/kg/week) as an intraperitoneal injection. Gated ^99 m^Tc-MIBI SPECT was performed for cardiac function evaluation, and ^99 m^Tc-DSMA scintigraphy was performed for renal function assessment. Various organs’ uptake (%ID/g) of ^125^I-OI5V was estimated in the rats before and at three, five, and seven weeks after the injection.

**Results:**

No rats died during DOX injections, until eight weeks. The left ventricular cavity volume increased compared to before DOX injection at five weeks after DOX injection. At seven weeks post-DOX injection, the ejection fraction decreased compared with that before the injection. DMSA scintigraphy revealed that renal function decreased significantly after seven weeks. In the post-mortem tissue counting study, ^125^I-OI5V uptake decreased from five weeks post-injection. After DOX injection, the tracer uptake in the kidney decreased and the tracer uptake in the blood increased.

**Conclusion:**

The present study confirmed the expression pattern of σ1R expression after DOX injection. A decrease in σ1R expression detected using ^125^I-OI5V may serve as an earlier marker of DOX-induced cardiotoxicity compared with ejection fraction decline.

## Introduction

Doxorubicin (DOX) is a widely used chemotherapeutic agent; however, its use is often limited by chemotherapy-induced cardiotoxicity, a major concern for patients undergoing tumor treatment. Drug-induced cardiotoxicity represents a significant obstacle in cancer therapy, as it frequently interferes with the continuation of life-saving treatment options. Cardiotoxicity has been reported with several drugs, including cisplatin, trastuzumab, rosiglitazone, pioglitazone, and zidovudine. Numerous studies have identified diverse molecular mechanisms underlying drug-induced cardiomyocyte damage and death, including endoplasmic reticulum (ER) stress, oxidative stress (OS), disruptions in iron metabolism and Ca^2^⁺ homeostasis, sarcomeric structure alterations, gene expression modulation, and apoptosis [1]. Clinically, cardiotoxicity manifests as increased cardiac biomarkers, structural myocardial deformation, and reduced left ventricular ejection fraction (LVEF) [2, 3]. The widely accepted definition of cardiotoxicity is the decline in ejection fraction [2] of ≥ 10% to < 55% [4, 5].

The sigma-1 receptor (σ1R), first cloned in 1996 from guinea pig liver [6], is primarily localized to the ER membrane at mitochondria-associated membranes [7] and translocates to the plasma membrane under stress. As a chaperone protein, σ1R plays a crucial role in regulating protein folding and degradation, ER/OS stress responses, and cell survival [8]. It is recognized as a pluripotent modulator with functional implications across the living system [9]. While extensively studied in the central nervous system, σ1R has also been implicated in conditions such as myocardial hypertrophy, myocardial ischemia, and cancer [10–12].

In the heart, σ1R is expressed in cardiomyocytes, where it acts as a chaperone to regulate ER stress responses, calcium handling, and voltage-gated ion channel functions under pathological conditions [8]. σ1R has been shown to elucidate the pathophysiology of myocardial hypertrophy. For example, in transverse aortic constriction [13] mouse models, σ1R expression is downregulated in cardiomyocytes during heart failure [14]. However, the spatiotemporal expression patterns of σ1R in the context of DOX-induced cardiotoxicity (DIC) have not yet been investigated.

The radiotracer ^125^I-2-[4-(2-iodophenyl)piperidino]cyclopentanol (OI5 V), a vesamicol analog with a five-membered ring structure, exhibits a highly selective binding affinity for σ1R and shows promise as an imaging probe for σ1R [15]. In this study, we evaluated the spatiotemporal changes in σ1R expression, using ^125^I-OI5 V, along with cardiac function assessments via ^99 m^Tc-methoxyisobutylisonitrile (MIBI) single-photon emission computed tomography (SPECT) in a rat model of DIC. In addition, renal function was assessed via ^99 m^Tc-DMSA to explore the systemic impact of DIC on σ1R expression.

## Materials and methods

### Animal model of DOX cardiotoxicity

All experimental animal protocols were approved by the Institute for Animal Studies at Kanazawa University. Male Wistar rats (eight weeks old) were administered low-dose DOX (doxorubicin hydrochloride, NIPPON KAYAKU, Tokyo, Japan) intraperitoneally at 2 mg/kg weekly [16]. ^99 m^Tc- hexakis 2-methoxyisobutyl isonitrile (MIBI) SPECT imaging and post-mortem tissue counting were performed after the DOX injection.

### ***In vivo*** gated ^99 m^Tc- MIBI SPECT imaging

All rats underwent SPECT imaging on a dedicated small-animal SPECT system (U-SPECT, MILabs, Utrecht, The Netherlands) equipped with interchangeable multi-pinhole collimators**.** A dose of 185 MBq of ^99 m^Tc-MIBI (PDRadiopharma Inc., Tokyo, Japan) was injected via the tail vein 30 min before imaging. Scans were conducted under 1%–2% isoflurane anesthesia for 15 min. Gated SPECT imaging was performed at baseline and at three, five, seven, and eight weeks following DOX injection.

### SPECT data

SPECT data were acquired in the list mode, with photopeak windows centered at 140 keV (20% width). The triple-energy window scatter correction was applied using adjacent windows of 4.5% width. Data were reconstructed using pixel-based ordered-subset expectation maximization with 13 subsets and six iterations, without attenuation correction [17, 18].

The voxel size for ^99 m^Tc-MIBI SPECT was set to 0.8 × 0.8 × 0.8 mm and magnified by a factor of 10 to match the human reference heart resolution. Gated SPECT data were quantitatively analyzed using the QGS software to calculate the end-diastolic volume (EDV), end-systolic volume (ESV), and EF [2]. Each parameter’s normal reference value is shown in Table 1, and these values are based on the previous research findings of Hiromasa et al. [19].

**Table 1:** Normal reference values for BW, HR, EDV, ESV, SV, and EF [19]

Ages (weeks)	BW (g)	HR (bpm)	EDV (μL)	ESV (μL)	SV (μL)	EF (%)
8	233 ± 2	378 ± 39	316 ± 26	114 ± 8	202 ± 21	64.0 ± 2.2
9	276 ± 7	410 ± 18	349 ± 24	131 ± 11	218 ± 21	62.3 ± 3.1
10	312 ± 9	406 ± 24	393 ± 16	145 ± 20	248 ± 10	63.2 ± 3.8
12	358 ± 12	399 ± 23	434 ± 37	168 ± 17	266 ± 24	61.3 ± 2.1
16	415 ± 15	409 ± 16	479 ± 35	203 ± 14	276 ± 25	57.7 ± 1.8
21	450 ± 15	391 ± 20	530 ± 52	240 ± 21	290 ± 43	54.3 ± 4.1
25	471 ± 18	377 ± 14	497 ± 51	221 ± 35	277 ± 26	55.8 ± 3.7
28	492 ± 19	396 ± 22	503 ± 43	227 ± 11	276 ± 35	54.8 ± 2.7

*BW* body weight; *HR*, heart rate, *EDV* left ventricular end-diastolic volume, *ESV* left ventricular end-systolic volume, *SV* stroke volume, *EF* ejection fraction

### ^99 m^Tc-DMSA static scintigraphy

The acquired dimercaptosuccinic acid (DMSA) (PDRadiopharma Inc., Tokyo, Japan) images were analyzed using the public domain AMIDE imaging software (version 1.01). Regions of interest (ROIs) were manually delineated for the right and left kidneys. The mean radioactivity concentration within each ROI was measured as the percentage of the injected dose per cubic centimeter (%ID/cm^3^). The total %ID for both kidneys was then averaged to provide a combined measurement.

### Radiolabeling of ^125^I-OI5 V

^125^I-OI5 V was synthesized from 2-[4-(2-trimethylstannylphenyl)piperidino]cyclopentanol (OT5 V, 50 µg/50 µL) and ^125^I-NaI (37 MBq) via an iodo-destannylation reaction under no-carrier-added conditions [15]. After incubation for 10–20 min at room temperature, the product was purified via high-performance liquid chromatography (HPLC) using a reverse-phase HPLC column (Zorbax-ODS RX-C18, 9.6 × 250 mm) at 40 °C with a flow rate of 2.0 mL/min and a mobile phase of 80:20:0.2 v/v/v acetonitrile/H2O/monoethanolamine. The radiochemical yield was 70–80%, and the final radiochemical purity exceeded 99%, as confirmed by analytical radio-HPLC analysis.

### Post-mortem tissue counting

Rats received ^125^I-OI5 V (0.25 MBq) via tail vein injection at zero, three, five, seven, and eight weeks after DOX administration (n = 4–6 per group). Thirty minutes after injection, the rats were euthanized, and the organs of interest (blood, lung, heart, liver, kidney, spleen, pancreas, stomach, intestine, muscle, and bone) were harvested, weighed, and analyzed for radioactivity using an automated gamma counter. Tissue radioactivity concentrations were expressed as the percentage of the injected dose per gram of tissue (%ID/g).

### Immunohistochemical analysis and histological staining

Heart tissues from zero, five, and eight weeks were fixed in 4% paraformaldehyde and embedded in paraffin. Left ventricular short-axis Sects. (5 µm thick) were prepared from the midventricular region. Sections were stained with an anti-transforming growth factor (TGF) -β1 antibody (ab215715, Abcam) using a direct immunoperoxidase method (ab64261, Abcam), followed by hematoxylin counterstaining for nuclear visualization. Masson’s trichrome staining (Trichrome Stain Kit, ScyTek Laboratories, Inc., USA) was performed as per the manufacturer’s protocol to assess fibrosis. TGF-β1 expression and fibrosis were examined using a fluorescence microscope (BZ-X800, Keyence, Osaka, Japan), and the area ratio of TGF-β1-positive regions was measured using ImageJ (version 1.54 k).

### Statistical analysis

Statistical analyses were performed using JMP® (SAS Institute, Cary, NC, USA). Quantitative data are presented as the mean ± standard deviation, while categorical data are presented as frequencies with percentages. Group comparisons were performed using the analysis of variance (ANOVA) and Tukey’s honestly significant difference test. A P-value < 0.05 was considered statistically significant.

## Results

### Body weight

The mean body weight of the rats was 260 ± 10 g before DOX injection. This parameter progressively increased, rising to 319 ± 17 g at 3 weeks, 326 ± 18 g at five weeks, 330 ± 17 g at seven weeks, and 342 ± 23 g at eight weeks. The detailed results are summarized in Table 2.

**Table 2:** Body weight after doxorubicin injection

	0 weeks	3 weeks	5 weeks	7 weeks	8 weeks	P-value of ANOVA
BW (g)	260 ± 10	319 ± 17*	326 ± 18**	330 ± 17***	342 ± 21****^, +^	< 0.0001

^*^0 wk vs. 3wk, p < 0.05

^**^0 wk vs. 5wk, p < 0.05

^***^0 wk vs. 7wk, p < 0.05

^****^0 wk vs. 8wk, p < 0.05

^+^3 wk vs. 8 wk, p < 0.05 BW, body weight

### Cardiac function by gated ^99 m^Tc-MIBI SPECT/CT

Cardiac function was evaluated using gated ^99 m^Tc-MIBI SPECT imaging. At five weeks post-DOX injection, an increase in the left ventricular cavity volume was observed relative to the baseline (pre-DOX injection) value. The results highlight the comparatively progressive cardiac dysfunction following DOX administration. A detailed summary of the post-mortem tissue counting results is presented in Table 3.

**Table 3:** Cardiac function evaluated by gated ^99 m^Tc-MIBI SPECT after doxorubicin injection

	0 weeks	3 weeks	5 weeks	7 weeks	8 weeks	P-value of ANOVA
EDV(μL)	323 ± 20	352 ± 39	395 ± 13 *	414 ± 49**^, +^	421 ± 50 ***	< 0.001
ESV(μL)	114 ± 11	141 ± 13	167 ± 20 *	196 ± 24**^, +,/^	221 ± 22***^, ++,//^	< 0.0001
EF(%)	65 ± 3	59 ± 7	57 ± 5	52 ± 6 **	47 ± 8***^, ++,//^	< 0.0001

^*^0 wk vs. 5wk, p < 0.05

^**^0 wk vs. 7wk, p < 0.05

^***^0 wk vs. 8wk, p < 0.05

^+^3 wk vs. 7wk, p < 0.05

^++^3 wk vs. 8wk, p < 0.05

^/^5 wk vs. 7wk, p < 0.05

//5 wk vs. 8wk, p < 0.05

*EDV* left ventricular end-diastolic volume, *ESV* left ventricular end-systolic volume, *EF* ejection fraction

### ^99 m^Tc-DMSA renal scintigraphy

**Fig. 1:**
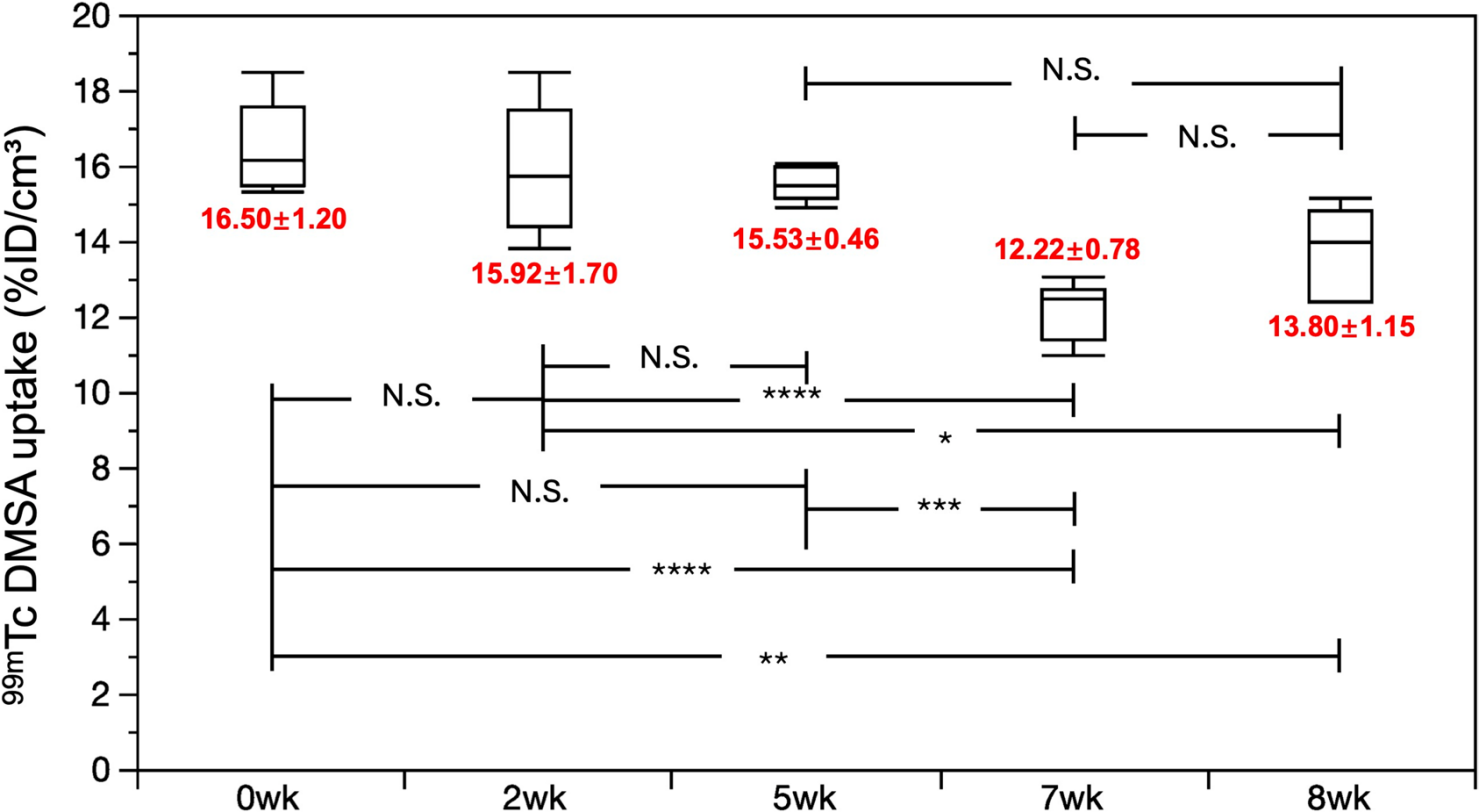
Average Bilateral Renal DMSA Uptake and Functional Assessment. Statistical comparisons between time points (*P < 0.05, **P < 0.01, ***P < 0.001, ****P < 0.0001) are noted in the Figure. N.S., no significance; DMSA, dimercaptosuccinic acid

^99 m^Tc-DMSA scintigraphy was used to evaluate the function of both kidneys. The averaged renal uptake of 99 mTc-DMSA demonstrated a significant decline in renal function starting from the seventh week post-DOX injection. Detailed data are presented in Fig. 1.

### Immunohistochemical analysis and histological staining

**Fig. 2:**
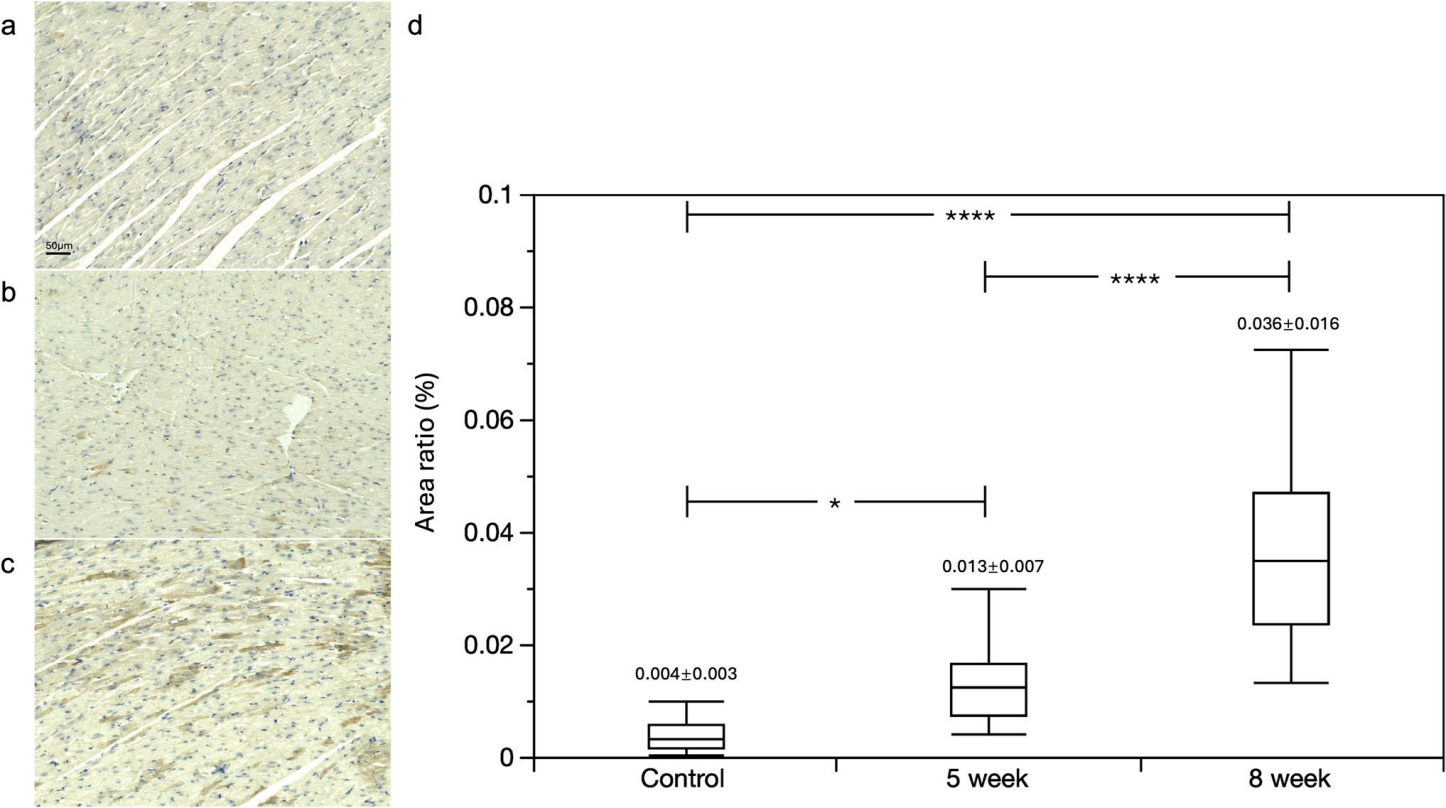
Area Ratio of TGF beta 1 to the LV area. Representative immunohistochemically stained images of the control group (**a**), five-week group (**b**), and eight-week group (**c**). Scale bars = 50 μm. Immunohistochemical staining (brown) demonstrates an increase in TGF-β1 expression in the myocardium over time. **d** Time course analysis of the TGF-β1-positive area ratio relative to the left ventricular (LV) area. The box-and-whisker plots represent the interquartile range (25%–75%), with the horizontal line indicating the median value. The whiskers denote the upper and lower range limits. A significant increase in TGF-β1 expression is observed from five weeks onward compared with the control group, persisting through eight weeks. TGF, transforming growth factor; *p < 0.05, ****p < 0.0001

**Fig. 3:**
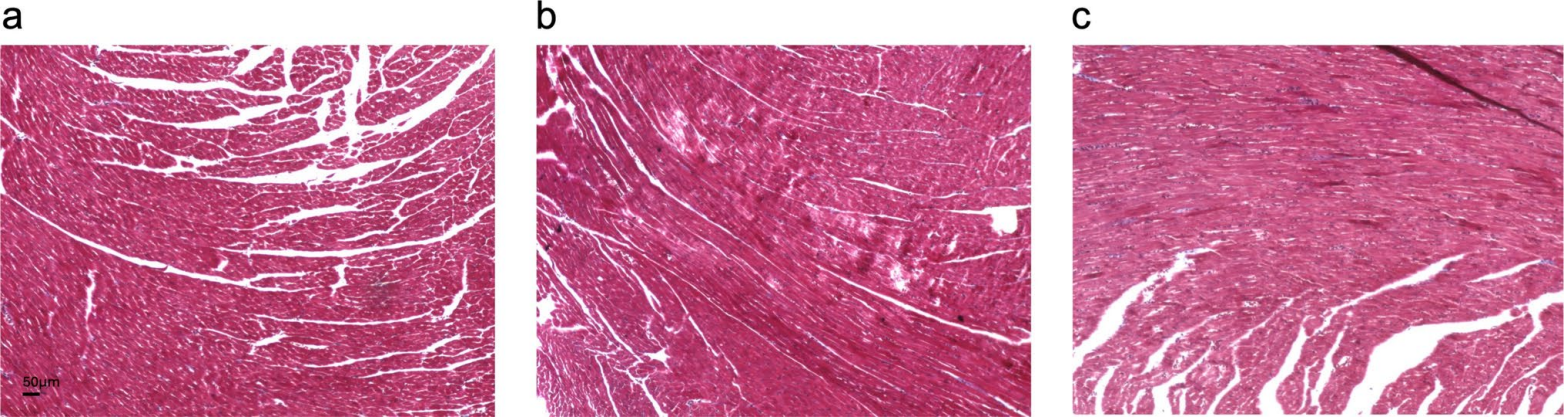
Masson’s trichrome staining revealed no significant visible differences. Fibrosis in the myocardium is displayed in blue in Masson’s trichrome staining. Representative histological staining images of the control group **a**, the five-week group **b**, and the eight-week group **c**. Scale bars = 50 μm

Immunohistochemical staining demonstrated a significant increase in TGF-β1 expression in the myocardium at five weeks post-DOX injection, which further intensified at eight weeks (Fig. 2). This finding indicates a progressive upregulation of TGF-β1 in response to DOX treatment, suggesting its potential involvement in myocardial remodeling and fibrosis. However, no significant differences in fibrosis were detected based on Masson’s trichrome staining (Fig. 3).

### Systemic ^125^I-OI5 V distribution

**Fig. 4:**
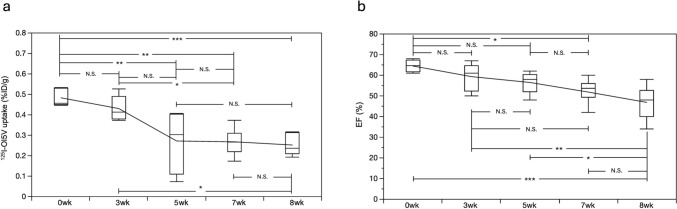
Early Reduction in Cardiac ^125^I-OI5 V Uptake Compared to the Ejection Fraction. The box plot for.^125^I-OI5 V uptake is shown in **a**, with the median values connected by a line. The box plot for EF% is displayed in **b**, with the median values connected by a line. EF, ejection fraction; N.S., no significance; *p < 0.05, **p < 0.01, ***p < 0.001

The in vivo biodistribution of ^125^I-OI5 V was assessed 30 min after tracer administration. Before and after DOX injection, high tracer uptake was observed in the lung, kidney, and pancreas, whereas tracer uptake in the blood remained consistently low. However, following DOX injection, tracer uptake in the kidney significantly decreased, whereas uptake in the blood increased. Additionally, cardiac tracer uptake showed a significant decline five weeks after DOX injection. These results suggest an altered systemic distribution of ^125^I-OI5 V following DIC. A detailed summary of the post-mortem tissue counting results is provided in Table 4. The temporal trends in EF% and cardiac ^125^I-OI5 V uptake are summarized in Fig. 4, illustrating that a significant reduction in cardiac ^125^I-OI5 V uptake occurs earlier than the decline in EF%, reinforcing its potential as an early marker for DOX-induced cardiotoxicity.

**Table 4:** Post-mortem tissue counting in rats at 30 min after ^125^I-OI5 V injection

Organ	Uptake (%ID/g)					P-value of ANOVA
	0 weeks	3 weeks	5 weeks	7 weeks	8 weeks	
Blood	0.053 ± 0.006	0.064 ± 0.009	0.078 ± 0.008 *	0.072 ± 0.006 **	0.070 ± 0.009 ***	< 0.001
Heart	0.48 ± 0.04	0.43 ± 0.06	0.27 ± 0.15 *	0.27 ± 0.07**^, +^	0.25 ± 0.05***^, ++^	< 0.0001
Lung	2.13 ± 0.38	2.36 ± 1.01	1.40 ± 0.51	1.56 ± 0.43	1.81 ± 0.91	N.S
Kidney	1.99 ± 0.13	1.31 ± 0.83	0.91 ± 0.47 *	1.15 ± 0.26 **	1.19 ± 0.30	< 0.05
Pancreas	2.04 ± 1.33	1.83 ± 0.87	1.31 ± 0.64	1.61 ± 0.40	1.46 ± 0.32	N.S
Muscle	0.09 ± 0.01	0.07 ± 0.02	0.07 ± 0.05	0.06 ± 0.01	0.07 ± 0.01	N.S
Liver	1.07 ± 0.13	1.57 ± 0.85	0.94 ± 0.37	1.09 ± 0.33	0.76 ± 0.16	N.S

^*^0 wk vs. 5wk, p < 0.05

^**^0 wk vs. 7wk, p < 0.05

^***^0 wk vs. 8wk, p < 0.05

^+^3 wk vs. 7wk, p < 0.05

^++^3 wk vs. 8wk, p < 0.05

## Discussion

The present study demonstrated a significant decrease in σ1R expression following weekly DOX injections. To our knowledge, this is the first study to report such findings. Post-mortem tissue analyses revealed that the decline in σ1R occurred 5 weeks earlier than the reduction in LVEF at seven weeks. These findings suggest that in the DOX toxicity model, σ1R expression decreases earlier than functional changes occur in the heart. Furthermore, imaging revealed dynamic changes in σ1R expression in response to DOX, which highlights its potential as a biomarker for early myocardial injury.

In addition, our current imaging approach assessed global cardiac σ1R changes and cannot rule out regional differences in σ1R downregulation within the myocardium. Previous studies have reported preferential impairment in the basal segments of the left ventricle following DOX exposure [20], suggesting that specific myocardial regions may be more susceptible to σ1R loss. To elucidate potential spatial variations in σ1R expression, future investigations should incorporate high-resolution small-animal imaging or ex vivo autoradiography, which are capable of mapping regional σ1R distribution and evaluating localized receptor downregulation associated with DOX-induced cardiotoxicity.

Notably, our immunohistochemical analysis also confirmed an increase in TGF-β1 expression in the myocardium, coinciding with the observed σ1R downregulation. This relationship raises important questions about the potential relationship between TGF-β1 upregulation and σ1R loss in the pathophysiology of DOX cardiotoxicity. While σ1R has been well-documented to modulate ER stress, calcium handling, and oxidative stress in cardiomyocytes, its direct association with TGF-β1 expression in the myocardium requires further investigation [8–12].

Emerging evidence suggests that σ1R activity can directly influence fibrotic signaling. Chronic σ1R stimulation has been reported to protect against cardiac fibrosis by attenuating TGF-β1-driven cardiac fibroblast activation via PERK and IP3R-VDAC1-MCU signaling pathways, whereas downregulation of σ1R activity exacerbates myofibroblast activation and increases fibrotic markers [21–23]. While we did not observe overt fibrosis in the present study, the concurrent upregulation of TGF-β1 expression and downregulation of σ1R may reflect early activation of profibrotic pathways. These findings are consistent with an inverse relationship between σ1R signaling and TGF-β1 activity.

In this study, fibrosis did not increase significantly, despite the upregulation of TGF-β1 expression. TGF-β1 is known to be involved in fibroblast activation and driving excessive extracellular matrix deposition through both SMAD-dependent and non-SMAD pathways [24]. Considering the dose-dependent toxicity of DOX [25], this study employed a low-dose administration protocol, which did not induce significant myocardial fibrosis. The observed decline in cardiac function is likely associated with the upregulation of TGF-β1. Previous studies have indicated that TGF-β1 is not only involved in fibrosis but also contributes to ventricular arrhythmias [26], myocardial inflammatory responses [27], and the regulation of ion channel expression [28].

Sigma-1R is predominantly found in cardiomyocytes and plays a role in maintaining contractility and heart rate [29, 30]. Previous studies have highlighted σ1R's role in cardiac function preservation by modulating calcium handling, ion channel function, and apoptosis pathways [8, 31]. Our findings align with prior research showing σ1R downregulation in other models of cardiac dysfunction, such as transverse aortic constriction [13] [14]. The temporal precedence of σ1R reduction over LVEF decline further underscores the potential role of the former as an early marker of myocardial stress. Studies performed using σ1R ligands, agonists, or antagonists have provided insights into the downstream effects of σ1R modulation in the heart [9, 10, 23, 32–35]. As a chaperone protein, σ1R is implicated in regulating ER stress, calcium handling, voltage-gated ion channel function, cellular toxicity/apoptosis, and hypertrophy. Our findings further support the cardioprotective role of σ1R, although its precise involvement in the pathogenesis of DIC remains unclear. This study provides evidence of σ1R downregulation during DIC, complementing prior research on DOX-induced ER stress.

Furthermore, although this study did not include a separate untreated control group, each rat effectively served as its own control through baseline measurements. By comparing post-DOX values to the same animal’s baseline, we accounted for inter-animal variability and natural age-related changes. Additionally, our lab has previously collected baseline cardiac imaging data using the same rat strain, small-animal SPECT/CT system, and image reconstruction protocols, providing further reference values for normal physiology, as shown in Table 1 [19]. These prior data support the interpretation that the post-DOX changes are distinguishable from ordinary growth.

Importantly, although increases in total body weight could theoretically influence tracer distribution, the use of %ID/g inherently corrects for tissue mass. This value, defined as the radioactivity in a tissue divided by the product of the injected dose and tissue weight, is normalized to organ size and should not be confounded by systemic changes in body weight.

Interestingly, σ1R downregulation was also observed in the kidney at five weeks, consistent with the renal function impairment observed in the DOX toxicity model. While direct links between cardiac and renal σ1R downregulation remain unclear, systemic oxidative stress, ER stress, and inflammation could mediate σ1R loss in both organs.

Moreover, the reduced renal uptake of ^125^I-OI5 V may reflect not only σ1R downregulation but also DOX-induced nephrotoxicity leading to altered tracer pharmacokinetics [36]. Impaired renal function could diminish the renal clearance or retention of the tracer, resulting in higher circulating tracer levels and lower renal accumulation independent of σ1R expression changes. Previous studies have suggested that σ1R plays a protective role in renal ischemia models by mitigating ER-mitochondrial dysfunction [37, 38]. σ1R is a versatile modulator in various conditions, including ischemia [9]. In the brain, σ1R expression has been shown to increase in peri-infarcted areas following permanent middle cerebral artery occlusion [39]. These findings suggest that σ1R downregulation might reflect a broader systemic response to DOX toxicity, impacting multiple organ systems.

Although this study provides novel insights into σ1R’s role in DIC, several limitations should be acknowledged. First, the low-dose DOX model employed herein may not fully capture the spectrum of cardiotoxic effects observed at higher doses, potentially limiting the generalizability of the study’s findings. Future studies incorporating a wider range of DOX doses are warranted to better elucidate dose-dependent effects. Second, although each rat served as its own control based on baseline measurements, the absence of a parallel untreated cohort limits our ability to fully distinguish DOX-specific effects from those related to normal growth or procedural factors. Third, the lack of mechanistic experiments to directly link σ1R downregulation with TGF-β1-mediated fibrosis limits the study’s ability to establish causal relationships, as this study primarily focused on nuclear cardiology. Additionally, the mechanisms underlying fibroblast activation and the specific signaling pathways involving σ1R remain unclear, necessitating further functional assays at the cellular level. Lastly, the study did not explore potential therapeutic interventions targeting σ1R, which could have provided further insights into its protective role and translational potential.

## Conclusions

In conclusion, this study confirmed a spatiotemporal decline in σ1R expression following DOX administration, with σ1R downregulation preceding LVEF reduction. These findings highlight the potential of σ1R expression (which is detected via ^125^I-OI5 V imaging) as an early biomarker for DIC, offering diagnostic utility prior to observable EF decline. Further studies are called for to investigate the mechanistic interplay of σ1R in DIC progression and to explore potential therapeutic interventions aimed at mitigating DIC.

## Data Availability

The datasets generated during the current study are not publicly available due to ethical restrictions on animal research, but are available from the corresponding author on reasonable request.
